# Enalapril induced reversible acute renal failure detected by ^99m^Tc-DMSA renal scan in a patient with bilateral renal artery stenosis: a case report

**DOI:** 10.4076/1757-1626-2-7509

**Published:** 2009-09-09

**Authors:** Kianoush Ansari Gilani, Abbas Madani, Nahid Rahimzadeh, SeyedTaher Esfahani, Jamak Modaresi Esfeh

**Affiliations:** 1 Research Institute for Nuclear Medicine, Tehran University of Medical Sciences, Shariati HospitalNorth Kargar Ave. 14114, TehranIran; 2 Department of Nuclear Medicine, Dr. Gharib Ave. Keshavarz Blvd., Children’s Hospital Medical Center, Tehran University of Medical SciencesTehranIran; 3 Department of Pediatric Nephrology, Dr. Gharib Ave. Keshavarz Blvd., Children’s Hospital Medical Center, Tehran University of Medical Sciences14114, TehranIran

## Abstract

The authors report and discuss a case of bilateral renal artery stenosis in a hypertensive 9 year-old girl that was first suspected with ^99m^Technetium-dimercaptosuccinic acid renal scintigraphy. The scintigraphy showed signs of acute renal failure while the patient was on enalapril for controlling her hypertension. Bilateral renal artery stenosis was confirmed with computed tomography angiography. Hypertension resolved after bilateral renal artery angioplasty.

## Introduction

Renal artery stenosis (RAS) is a relatively common cause of secondary hypertension, accounting for 1% of unselected hypertensive children, but rises to as high as 10 to 40% in patients with severe or refractory hypertension [1,2]. Bilateral RAS or unilateral disease in a single kidney with acceptable GFR can be associated with persistent hypertension and progressive renal dysfunction [3]. Renovascular hypertension (RVH) can be asymptomatic or an incidental finding [3].

The blood pressure of the affected children can be controlled with combination of mild diuretic and angiotensin-converting enzyme (ACE) inhibitors or an angiotensin 2 receptor blocker (ARBs).

Captopril- or enalapril- enhanced renal scintigraphy with either ^99m^technetium-diethylene triamine penta-acetic acid (^99m^Tc-DTPA), ^99m^technetium-ethylenedicysteine (^99m^Tc-EC) or ^99m^technetium-mercapto acetyl triglycine (^99m^Tc-MAG3) is a widely accepted tool for the diagnosis of hemodynamically significant renal artery stenosis (RAS) [4,5]. Although not as popular as the above mentioned radiotracers, captopril- or enalapril-enhanced ^99m^Tc-DMSA is also used for the diagnosis of RAS with good sensitivity and specificity [6].

We are reporting a child with severe hypertension that was suspected to have bilateral RAS based on ^99m^Tc-DMSA findings. The diagnosis was confirmed by computed tomography (CT) angiography.

## Case presentation

**Figure 1.**
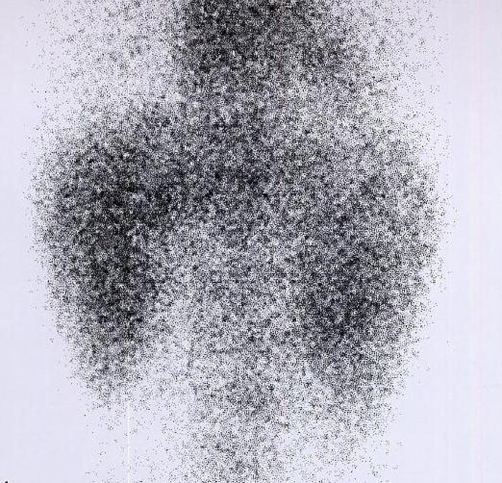
^99m^Tc-DMSA renal scintigraphy of the patient while on enalapril. The posterior image shows decreased radiotracer uptake in both kidneys. Radiotracer uptake in the liver and background is increased. This pattern is suggestive of renal failure.

We present a case of 9-year-old Caucasian girl from Iran admitted to the Children’s Hospital Medical Center for the evaluation of hypertension. Her problem was discovered at school in “blood pressure screening program” one month earlier. Her mother did not have any remarkable problem during pregnancy and delivery. The patient had normal neonatal period with acceptable growth and development during infancy and childhood. There was no history of hypertension in her family members. She did not have any remarkable complaints except episode of transient headache and nausea. On physical examination she was 25 kg and had a height of 123 cm. Her BP was 180/110 mmHg in the upper limbs, and 170/100 mmHg in the lower limbs. A pan-systolic grade 2/6 murmur on the left sternal border with no bruit at the abdomen was heard. Fundoscopic examination showed grade-4 hypertensive retinopathy. Mild cardiomegaly was evident on chest x ray and left ventricular hypertrophy (LVH) secondary to persistent arterial hypertension was found on echocardiography. Complete blood count, blood chemistry profile and urinalysis were in normal limits. On ultrasonography renal length was 66 mm and 81 mm in the right and left sides respectively and both kidneys showed normal echogenicity. As full doses anti-hypertensive treatment with different kinds of anti-hypertensive agents was unsuccessful enalapril was started with a dose of 0.1 mg/kg/day. The blood pressure was controlled after this drug regimen. The patient was referred to the nuclear medicine department for ^99m^TC-DMSA renal scintigraphy. The scintigraphy showed globally decreased cortical function of both kidneys with increased background activity and radiotracer uptake in the liver (Figure 1) which were suggestive of renal failure.

Considering the baseline normal serum creatinine level, treatment with enalapril and the scan pattern; possibility of enalapril- enhanced acute renal failure due to bilateral renal artery stenosis was suggested.

**Figure 2.**
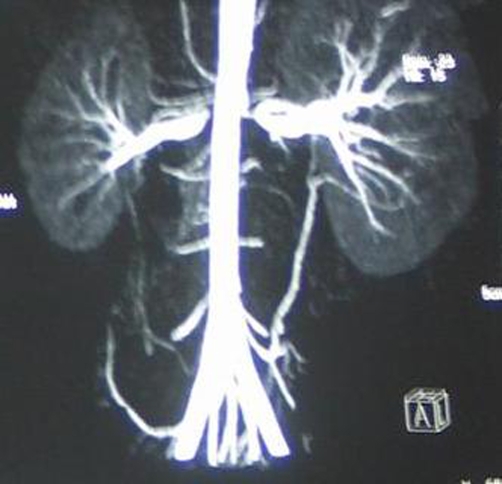
Computed tomography angiography of the renal arteries. Severe stenosis is visible in the proximal portions of the renal arteries bilaterally.

Further evaluation showed an increase in the level of serum BUN and creatinine from 18 to 114 mg/dl and 0.54 to 6.4 mg/dl respectively. Enalapril was stopped immediately and the patient was transferred to pediatric ICU. The levels of BUN and serum creatinine returned to pre-treatment values in the following 7 days. Patient underwent CT angiography for the evaluation of the renal arteries and confirmed the presence of bilateral renal artery stenosis (Figure 2).

**Figure 3.**
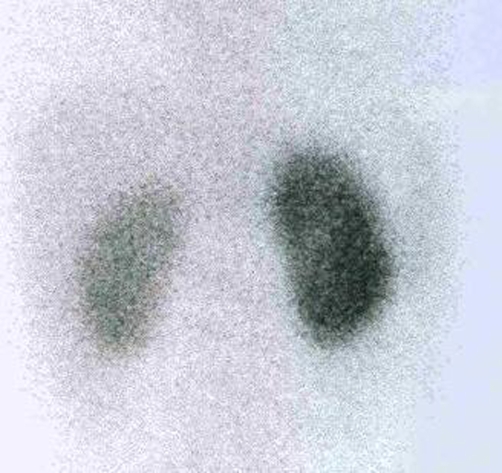
Follow-up ^99m^TC-DMSA renal scintigraphy after enalapril was discontinued. The posterior image shows considerable improvement in the cortical uptake of both kidneys. Right kidney shows normal cortical function, but the left kidney is smaller than the contralateral kidney and its cortical function is less than optimal.

Follow-up DMSA renal scan after 2 weeks of enalapril discontinuation showed marked improvement in the renal function with decrease in background activity and liver uptake. Although the cortical function of both kidneys was significantly improved, the left kidney function failed to show complete return to normal (Figure 3). Patient’s blood pressure returned to normal after bilateral renal artery angioplasty.

## Discussion

Systemic hypertension is less common in children than in adults but the incidence of hypertension in children is reported to be as high as 1-5% [1]. Unlike adults,70-80% of hypertension in children may have secondary and correctable etiologies [1]. More than half of hypertensive children are asymptomatic and this problem may be discovered during a routine examination. Others may come to clinical attention only after critical situations such as pulmonary edema, hypertensive encephalopathy and oliguric renal failure [1]. Similar to other reports [7], using enalapril in our patient with the diagnosis of RVH resulted in a dramatic decrease in glomerular filtration rate (GFR).In patients with unilateral RAS and a normal contralateral kidney no clinically detectable change in renal function is observed due to the effective compensation of the normal kidney [7]. However in cases with bilateral RAS or those with solitary kidney (where ineffective intraglomerular shunting of blood takes place and no reserve capacity exist), an increase in serum creatinine and deterioration in renal function is predictable after ACE inhibitor therapy[7]. These phenomena have potential clinical relevance in selecting appropriate therapy for patients with RVH. Patients who suffer from acute deterioration of renal function when exposed to ACE-inhibitors should be evaluated for the presence of either a solitary kidney with RAS or bilateral RAS. Stopping the ACE inhibitor agents is generally followed by improvement of renal function [7]. Either angioplasty or surgery is recommended in patients who show severe decline in GFR after using antihypertensive agents or those who fail to show long term BP control and stability of renal function [8].

Captopril- or enalapril- enhanced renal scintigraphy with ^99m^Tc-DTPA, ^99m^Tc-EC or ^99m^Tc-MAG3 is a widely accepted tool for the diagnosis of hemodynamically significant renal artery stenosis in cases with high pretest probability of secondary hypertension [4,5]. Although ^99m^Tc-DMSA renal scintigraphy is mostly used in the diagnosis of acute pyelonephritis and post-infection renal scars [9], the clinical use of captopril- or enalapril-enhanced ^99m^Tc-DMSA renal scan for the diagnosis of RAS (as in this case) is well documented with reasonably good sensitivity and specificity [6]. In this case possibility of bilateral RAS was first suggested by ^99m^Tc-DMSA renal scan and later was confirmed with other imaging modalities.

## Abbreviations

BUN: blood urea nitrogen
CT: computed tomography
^99m^Tc-DMSA: ^99m^Technetium-dimercaptosuccinic acid
^99m^Tc-DTPA: ^99m^technetium-diethylene triamine penta-acetic acid
^99m^Tc-EC: ^99m^technetium-ethylenedicysteine
^99m^Tc-MAG3: ^99m^technetium-mercapto acetyl triglycine
